# Silybin-Mediated Inhibition of Notch Signaling Exerts Antitumor Activity in Human Hepatocellular Carcinoma Cells

**DOI:** 10.1371/journal.pone.0083699

**Published:** 2013-12-27

**Authors:** Song Zhang, Yang Yang, Zhenxing Liang, Weixun Duan, Jian Yang, Juanjuan Yan, Ning Wang, Wenqiang Feng, Meiling Ding, Yongzhan Nie, Zhenxiao Jin

**Affiliations:** 1 State Key Laboratory of Cancer Biology, Department of Gastroenterology, Xijing Hospital, The Fourth Military Medical University, Xi’an City, China; 2 Department of Cardiovascular Surgery, Xijing Hospital, The Fourth Military Medical University, Xi’an City, China; 3 Department of Prosthodontics, School of Stomatology, The Fourth Military Medical University, Xi’an City, China; University of Hong Kong, Hong Kong

## Abstract

Hepatocellular carcinoma (HCC) is a global health burden that is associated with limited treatment options and poor patient prognoses. Silybin (SIL), an antioxidant derived from the milk thistle plant (*Silybum marianum*), has been reported to exert hepatoprotective and antitumorigenic effects both in vitro and in vivo. While SIL has been shown to have potent antitumor activity against various types of cancer, including HCC, the molecular mechanisms underlying the effects of SIL remain largely unknown. The Notch signaling pathway plays crucial roles in tumorigenesis and immune development. In the present study, we assessed the antitumor activity of SIL in human HCC HepG2 cells in vitro and in vivo and explored the roles of the Notch pathway and of the apoptosis-related signaling pathway on the activity of SIL. SIL treatment resulted in a dose- and time-dependent inhibition of HCC cell viability. Additionally, SIL exhibited strong antitumor activity, as evidenced not only by reductions in tumor cell adhesion, migration, intracellular glutathione (GSH) levels and total antioxidant capability (T-AOC) but also by increases in the apoptotic index, caspase3 activity, and reactive oxygen species (ROS). Furthermore, SIL treatment decreased the expression of the Notch1 intracellular domain (NICD), RBP-Jκ, and Hes1 proteins, upregulated the apoptosis pathway-related protein Bax, and downregulated Bcl2, survivin, and cyclin D1. Notch1 siRNA (in vitro) or DAPT (a known Notch1 inhibitor, in vivo) further enhanced the antitumor activity of SIL, and recombinant Jagged1 protein (a known Notch ligand in vitro) attenuated the antitumor activity of SIL. Taken together, these data indicate that SIL is a potent inhibitor of HCC cell growth that targets the Notch signaling pathway and suggest that the inhibition of Notch signaling may be a novel therapeutic intervention for HCC.

## Introduction

Hepatocellular carcinoma (HCC) is currently the fifth most common cancer and the third leading cause of cancer-related deaths worldwide; over 600,000 patients die as a result of liver cancer annually. Despite significant advances in surgery and chemotherapy, the majority of patients with HCC die within one year of diagnosis [1]. In addition, these treatment methods are often associated with side effects and inadequately treat the disease. Thus, new treatment options are desperately needed. Despite the emergence of novel targeted agents and the use of various therapeutic combinations, no curative treatment options currently exist for patients with advanced cancer. The magnitude of this problem mandates the development of novel therapeutic agents, specifically, chemopreventive agents that are generated from less harmful natural materials [2], [3].

The flavonolignan silybin (SIL) constitutes the major biologically active component ofsilymarin extract, which was isolated from the milk thistle plant (Silybum marianum) [4]. Milk thistle extract has been used as a hepatoprotective substance for more than 2,000 years and is known to be non-toxic [5]. Over the last decade, numerous studies have shown that its main component, SIL, exhibits anticancer and chemopreventive properties in various in vitro and in vivo models of various cancers, including lung [5], colorectal [6], breast [7], prostate [7], brain [8], ovarian [9], and kidney [10] cancers. With respect to hepatocellular carcinoma (HCC), SIL has been implicated in significant growth inhibition and apoptosis in both HepG2 and PLC/PRF/5 HCC cells [11], [12]. However, the mechanisms underlying the anti-HCC effects of SIL have not been fully elucidated.

The Notch signaling pathway is highly conserved and regulates cell fate throughout embryonic development and adult life [13]. To date, four Notch receptors (Notch1–4) and two types of Notch ligands (Jagged1/2 and Delta1/3/4) have been discovered in mammals. The transcription factor RBP-Jκ, a well-known key component of the Notch signaling pathway, has been implicated in various cancers, including HCC [14]. Genes downstream of Notch in the signaling pathway include hairy and enhancer of split 1 (Hes1) and the hairy-related transcription (HRT) factor family [2]. The activation of Notch signaling can induce the expression of multiple targets that are involved in cellular proliferation, such as cyclin D1 and survivin [15]. Many studies have shown that Notch signaling is critical for physiologic angiogenesis. Notch signaling has also been implicated in tumor angiogenesis and metastasis [13]. Most importantly, Notch signaling has been reported to exert either oncogenic or tumor-suppressive functions in HCC tumorigenesis [16]. However, the role of Notch signaling in the antitumor activity of SIL has not been examined. In the present study, we assessed the antitumor activity of SIL in human HCC cells and explored the role of Notch signaling in SIL activity.

## Materials and Methods

### Materials

SIL, DAPT (a known Notch1 inhibitor), Notch1 siRNA and antibodies that had been raised against the Notch1 intracellular domain (NICD), Hes1, and RBP-Jκ were obtained from Santa Cruz Biotechnology (Santa Cruz, CA, USA). Antibodies that had been raised against survivin, cyclin D1, Bcl2, Bax, and β-actin were purchased from Cell Signaling Technology (Beverly, MA, USA). 3-(4,5-Dimethylthiazol-2-yl)-2,5-diphenyltetrazolium bromide (MTT), dithiothreitol (DTT), dimethyl sulfoxide (DMSO), and 2′,7′-dichlorofluorescein diacetate (2′,7′-DCFH-DA) were purchased from Sigma-Aldrich (St. Louis, MO, USA). Recombinant human Jagged1 protein was purchased from R&D Company (Minneapolis, MN, USA). The fluorescein isothiocyanate (FITC)-Annexin V/propidium iodide (PI) staining kit and Bradford protein assay kit were purchased from the Beyotime Institute of Biotechnology (Nanjing, Jiangsu, China). The glutathione (GSH) and total antioxidant capability (T-AOC) kits were obtained from the Nanjing Jiancheng Bioengineering Institute (Nanjing, Jiangsu, China). Rabbit anti-goat, goat anti-rabbit and goat anti-mouse secondary antibodies were purchased from the Zhongshan Company (Beijing, China).

### Cell Culture and Treatment

Human HCC HepG2 cells were obtained from the Cell Culture Center at the Chinese Academy of Medical Sciences (Shanghai, China). Cells were grown in Dulbecco’s modified Eagle medium (DMEM, Gibco, Carlsbad, CA, USA) supplemented with 10% fetal bovine serum (Gibco, Carlsbad, CA, USA), L-glutamine (2 mM), penicillin (100 units/ml), streptomycin (100 units/ml), and HEPES (25 mM). Cells were maintained in the presence of 5% CO_2_ at 37°C. Cells were treated with different concentrations of SIL (50, 100, and 200 µM dissolved in DMSO) for the indicated time periods. An equal amount of DMSO (vehicle) was present in each treatment, including the control; the DMSO concentration did not exceed 0.1% (v/v) in any of the treatments.

### Analysis of Cell Viability

An MTT assay was performed to assess the viability of the HepG2 cells. As described in our previous study [2], after the cells had been treated and washed with PBS, 100 µL of a 0.5 mg/mL MTT solution in phenol red-free DMEM was added to the cells, and the samples were incubated for 2 h at 37°C. Finally, 100 µL of DMSO was added to each well, and the samples were shaken for 15 min at 37°C. The results were analyzed at 490 nm using a SpectraMax 190 spectrophotometer (Molecular Devices, Sunnyvale, CA, USA), and cell viability was expressed in terms of the optical density (OD). In addition, cell morphology was observed under an inverted phase contrast microscope, and images were taken with a microscope (BX61, Olympus, Japan).

### Analysis of Cell Apoptosis

HepG2 cell apoptosis was detected using the FITC-Annexin V/PI staining kit. After SIL treatment, the cells were harvested, washed in ice-cold PBS, incubated for 15 min with fluorescein-conjugated Annexin V and PI, and analyzed using a FACScan flow cytometer equipped with the FACStation data management system and Cell Quest software (Becton Dickinson, San Jose, CA, USA).

### Analysis of Caspase3 Activity

As described previously [4], caspase3 activity was measured using a colorimetric assay kit (MBL International Corporation, Nagoya, Japan), according to the manufacturer’s recommended instructions. The cells were washed in ice-cold PBS, and the proteins were extracted and stored at −80°C. Cell lysates (20 µl) were added to a buffer that contained a p-nitroaniline (pNA)-conjugated substrate for caspase3 (Ac-DEVD-pNA) to yield a 100 µl total reaction volume. Incubations were performed at 37°C. The released pNA concentrations were calculated based on the absorbance values at 405 nm and the calibration curve of the defined pNA solutions. The caspase3 activity in the control group was set as 100%.

### Analyses of Intracellular Reactive Oxygen Species (ROS) Generation, GSH Levels, and T-AOC

The measurement of intracellular ROS was based on the ROS-mediated conversion of non-fluorescent 2′,7′-DCFH-DA into fluorescent DCFH. After treatment with SIL, the cells were trypsinized and subsequently incubated with DCFH-DA (20 µM) in PBS at 37°C for 2 h. After incubation, the DCFH fluorescence of the cells in each well was measured using an FLX 800 microplate fluorescence reader, with 530 nm as the emission wavelength and 485 nm as the excitation wavelength (Biotech Instruments Inc., USA). A cell-free condition was used to determine the background, and the fluorescence intensity in the control group was defined as 100%. Generation of intracellular reduced GSH, an index of the cellular reducing power, and T-AOC were measured using the appropriate kits according to the manufacturer’s recommended instructions. The GSH levels and T-AOC in the control group were both set to 100%.

### Analyses of Cell Adhesion and Migration

In our preliminary experiment, we found that SIL treatment (at concentrations of less than 20 µM) for 24 h had no effect on HepG2 cell proliferation. Therefore, we performed adhesion and migration assays with 24-h SIL treatments (5, 10, and 20 µM). These assays were performed as previously described [17]. After treatment with SIL, the cells were centrifuged and resuspended in basal medium containing 10% fetal bovine serum. Treated cells (1×10^4^ cells per well) were placed in a 96-well plate and incubated for 30 min at 37°C. After the cells were allowed to adhere for 30 min, they were gently washed 3 times with PBS. The adherent cells were stained with MTT and observed under an inverted phase contrast microscope. Pictures were taken using a BX61 camera (Olympus Company, Japan). Finally, 100 µL of DMSO was added to each well, and the samples were incubated for 15 min at 37°C with shaking. The wells were measured at 490 nm on a SpectraMax 190 spectrophotometer (Molecular Devices, Sunnyvale, CA, USA), and the OD value in the control group was set to 100%.

A cell culture wound-healing assay was performed to analyze cell migration. The cells were grown to confluence, and a linear wound was created in the confluent monolayer using a 200 µl micropipette tip. The cells were then washed with PBS to eliminate detached cells. After treatment with SIL(5, 10, and 20 µM) for 24 h, wound edge movement was monitored under a microscope. The results are expressed as the distance between the cells on either side of the scratch.

### Small Interfering RNA Transfection

For the siRNA transfections, HepG2 cells were plated into 6, 24, or 96-well plates and allowed to grow to subconfluency. The cells were transiently transfected with negative control siRNA or Notch1 siRNA at 50 pM for 48 h using the Lipofectamine RNAiMAX reagent (Invitrogen, Carlsbad, CA, USA) in OPTI-MEM media (Gibco, Carlsbad, CA, USA). The cells were subsequently prepared for use in further experiments.

### Antitumor Activity in a Xenograft Model

Male athymic nude mice were purchased from the Laboratory Animal Center of the Fourth Military Medical University. The mice were housed and maintained under specific pathogen-free conditions in facilities approved by the American Association for the Accreditation of Laboratory Animal Care and in accordance with the current regulations and standards of the United States Department of Agriculture, United States Department of Health and Human Services. The present study was performed according to the Guide for the Care and Use of Laboratory Animals, published by the US National Institutes of Health (National Institutes of Health Publication No. 85-23, revised 1996), and was approved by the Ethics Committee of the Fourth Military Medical University. All surgeries were performed under sodium pentobarbital anesthesia, and all efforts were made to minimize animal suffering. HepG2 cell tumor xenografts were established by subcutaneously injecting 1×10^6^ cells into the right flanks of 4 to 6-week-old male athymic nude mice. Based on the data from a pilot study, we initiated treatment when the tumor volumes reached approximately 100 mm^3^. The tumor volumes (V) were calculated with the following formula: V = A×B^2^/2 (A = largest diameter; B = smallest diameter). First, the mice were randomly divided into 3 groups (n = 6 per group): control (saline) and SIL at either 200 or 400 mg/kg body weight, 5 days/week, suspended in saline and fed by oral gavage. Next, the mice were randomly divided into control, SIL, DAPT+SIL, and DAPT groups (n = 6). SIL was orally administered to the mice at doses of 400 mg/kg body weight per day (5 days/week); DAPT (10 mg/kg) or control (0.05% DMSO) was diluted with saline and administered intraperitoneally (5 days/week). Tumor sizes were measured every 3 days using calipers (days 2, 5, 8, 11, 14, 17, and 20). On day 20, tumors were excised from euthanized mice for Western blot analysis.

### Western Blot Analyses

Cell samples were homogenized in lysis buffer containing 1% protease inhibitor cocktail. lysates were centrifuged for 15 min at 12,000×g, and the resulting supernatants were transferred to new tubes and stored at −70°C. Protein concentrations were determined using a Bradford protein assay kit, and proteins were separated using electrophoresis and transferred to nitrocellulose membranes. Membranes were blocked for 1.5 h in Tris-buffered saline and Tween-20 (TBST, pH 7.6) with 5% non-fat dry milk and were then incubated overnight at 4°C with antibodies that had been raised against Notch1, Hes1, RBP-Jκ (1∶500 dilution), survivin, cyclin D1, Bcl2, Bax, and β-actin (1∶1000 dilution), followed by washes with TBST. The membranes were then probed with the appropriate secondary antibodies (1∶5000 dilutions) at room temperature for 120 min and washed with TBST. Protein bands were detected using a Bio-Rad imaging system (Bio-Rad, Hercules, CA, USA) and quantified using the Quantity One software package (West Berkeley, CA, USA).

### Statistical Analyses

All of the experiments were performed in duplicate and repeated at least three times. The data are expressed as the means ± the standard error of the mean (SEM). The treatment groups were compared using one-way analysis of variance (ANOVA) in SPSS 12.0 (SPSS Inc., Chicago) software. Differences were considered statistically significant at P<0.05.

## Results

### Effects of SIL Treatment on HCC Cell Viability

**Figure 1:**
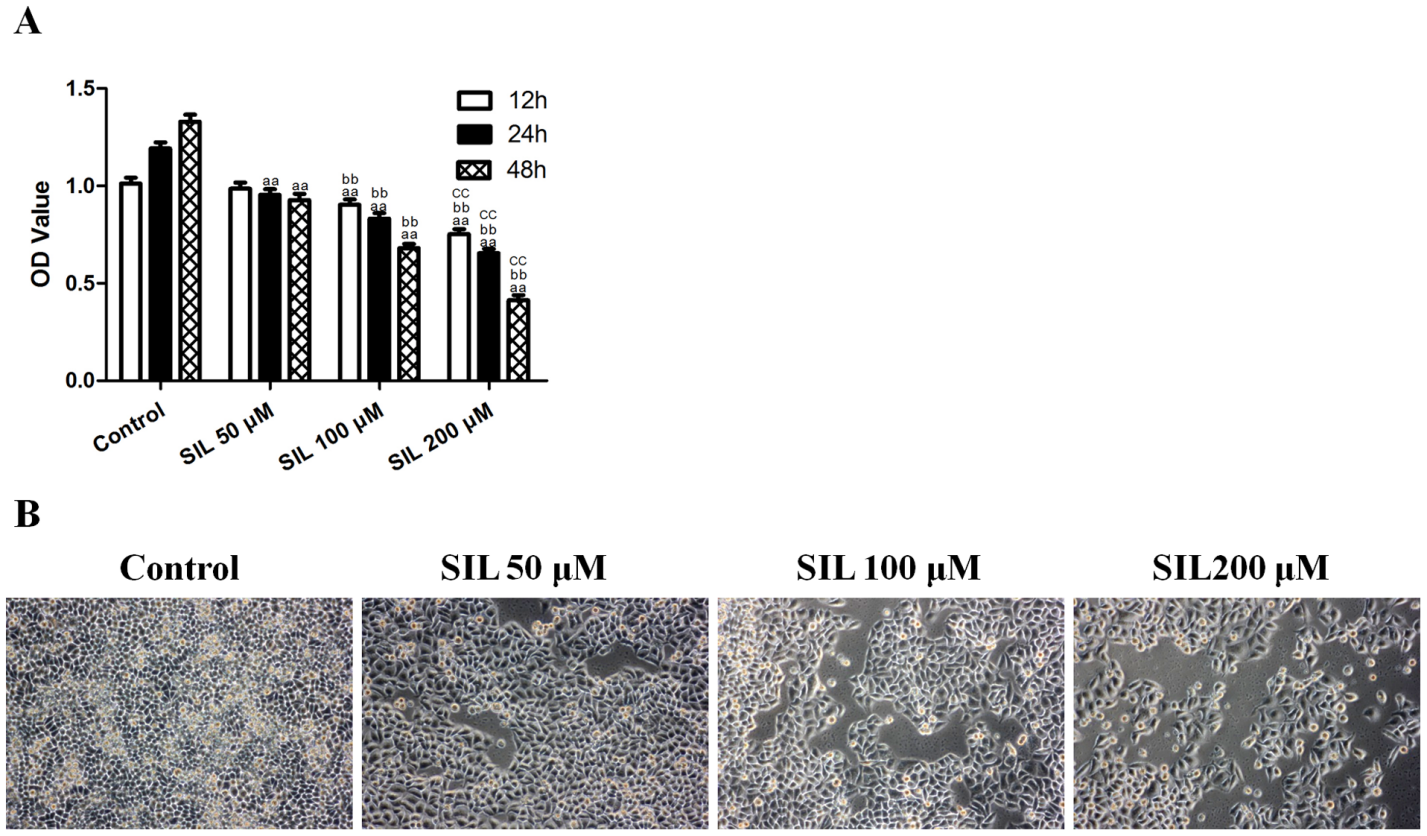
Effects of SIL treatment on HCC cell viability and morphology. **A.** Cells were treated with SIL at different concentrations (50, 100, and 200 µM) and assessed at different time points (12, 24, and 48 h). Viability is expressed as OD values. **B.** Cell morphology was observed under an inverted phase contrast microscope (after the cells had been treated for 24 h), and images were obtained. Significant cell shrinkage and a decreased cellular attachment rate were observed in the SIL-treated group. The results are expressed as the means ± SEM, n = 6. ^aa^P<0.01, compared with the control group, ^bb^P<0.01, compared with the 50 µM SIL-treated group, and ^cc^P<0.01, compared with the 100 µM SIL-treated group. SIL, silybin. OD, optical density.

The viability of SIL-treated HepG2 cells was determined using an MTT assay, and the data are presented in Figure 1A. Treatment of HepG2 cells for 12, 24, or 48 h with 50, 100, and 200 µM SIL inhibited cell viability in a dose- and time-dependent manner. Microscopy images (Figure 1B) indicated that SIL treatment resulted in significant cell shrinkage and decreased the rate of cellular attachment compared with the control group.

### Effects of SIL Treatment on HCC Cell Apoptosis

**Figure 2:**
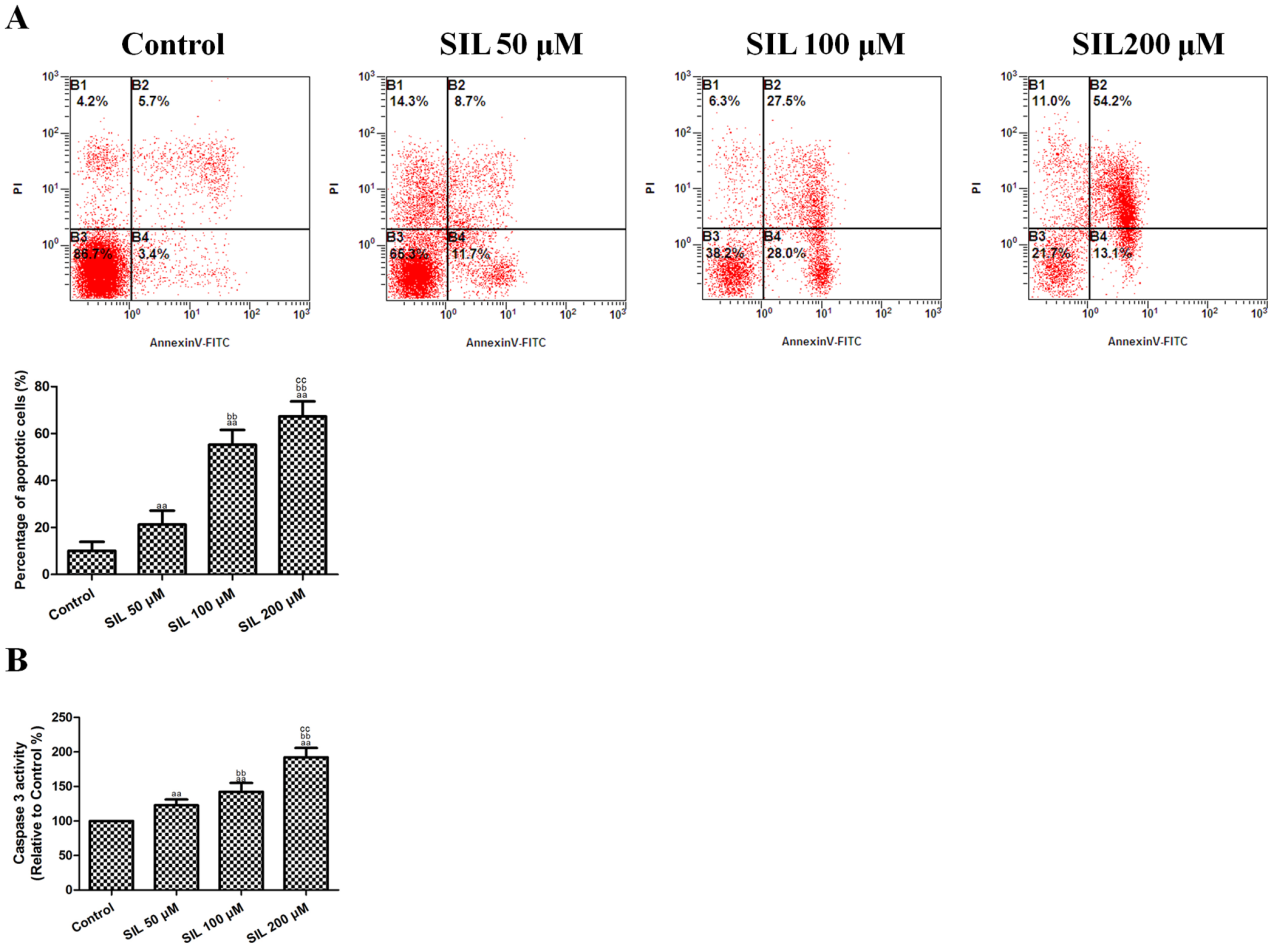
Effects of SIL treatment on the apoptotic rate and caspase3 activity in HCC cells. **A.** Representative flow cytometric analyses of apoptosis are shown. Four subpopulations and their fractions are indicated: normal cells (lower left), dead cells (upper left), early apoptotic cells (lower right), and late apoptotic cells (upper right). The apoptotic indices are expressed as the number of apoptotic cells/the total number of counted cells × 100%. **B.** The intracellular caspase3 activity levels are shown. The caspase3 activity level in the control group was defined as 100%. The results are expressed as the means ± SEM, n = 6. ^aa^P<0.01, compared with the control group, ^bb^P<0.01, compared with the 50 µM SIL-treated group, and ^cc^P<0.01, compared with the 100 µM SIL-treated group. SIL, silybin. OD, optical density.

The apoptotic index of SIL-treated HCC cells was also measured. After treatment with 50, 100, and 200 µM SIL for 24 h, the apoptotic index (Figure 2A) increased to 21.20±5.95%, 55.24±6.34%, and 67.29±6.41% (P<0.01, compared with the control group). Caspase3 activity (Figure 2B) also increased significantly to 122.80±8.26%, 142.30±12.77%, and 192.24±13.47% after SIL treatment (P<0.01, compared with the control group). Apoptosis and caspase3 induction were found to be dose-dependent. These results indicated that SIL induced apoptosis in HepG2 cells.

### Effects of SIL Treatment on HCC Cell Migration and Adhesion

**Figure 3:**
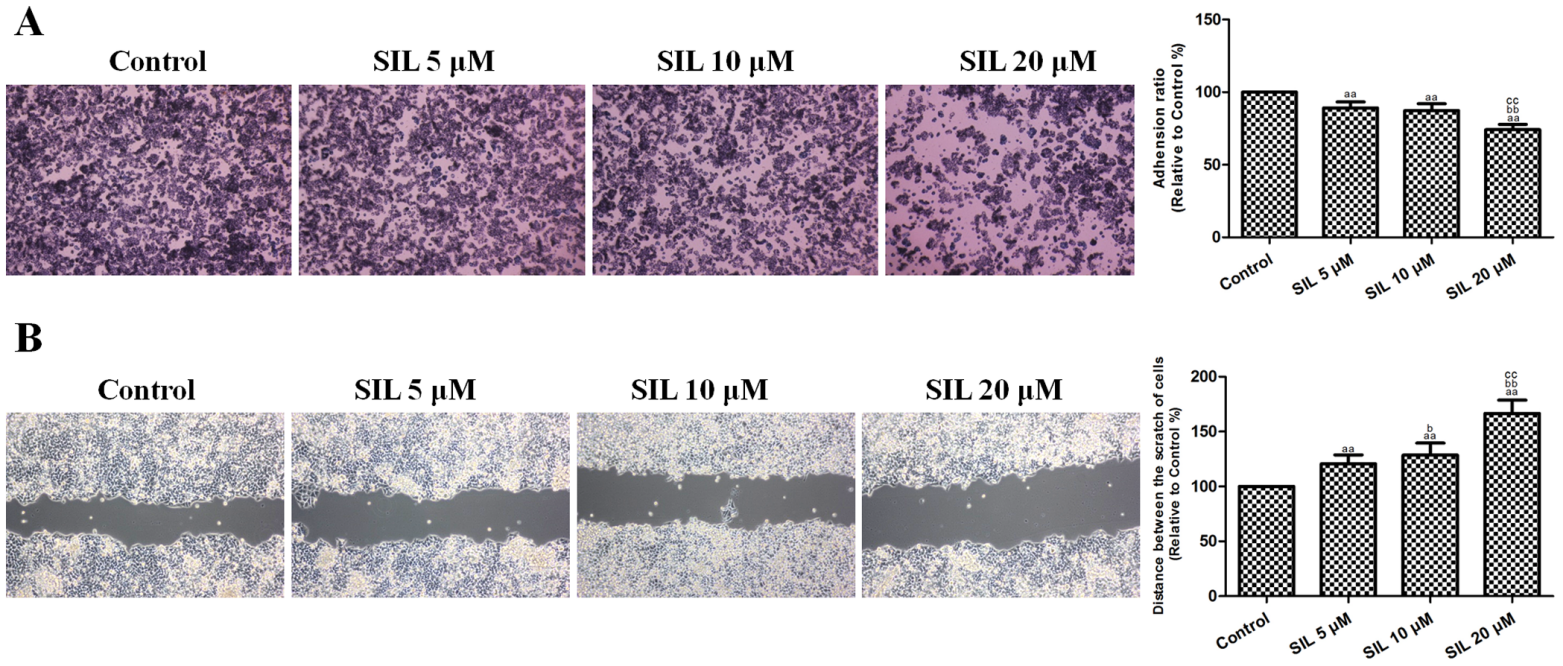
Effects of SIL treatment on HCC cell adhesion and migration (24 h). **A.** Representative adhesion images are shown. Cell adhesion ability is expressed as an adhesion ratio. The number of adherent cells in the control group was set to 100%. **B.** Representative wound healing images are shown. The migratory ability is expressed as the mean distance between the two sides of the scratch. The mean distance in the control group was set to 100%. The results are expressed as the means ± SEM, n = 6. ^aa^P<0.01, compared with the control group, ^bb^P<0.01, compared with the 5 µM SIL-treated group, and ^cc^P<0.01, compared with the 10 µM SIL-treated group. SIL, silybin.

The cell migration and adhesion of SIL-treated HCC cells were further evaluated. After incubation with SIL (5, 10, or 20 µM) for 24 h, the cell adhesion ratio decreased significantly to 89.22±4.10%, 87.35±4.71%, and 74.13±3.72% (P<0.01, compared with the control group, Figure 3A), and the distance between the scratches significantly increased to 120.63±8.19%, 128.60±10.92%, and 166.52±12.21% (P<0.01, compared with the control group, Figure 3B). These results indicated that SIL reduced the adhesive and migratory abilities of HepG2 cells.

### Effects of SIL Treatment on ROS Generation and GSH Levels in HCC Cells

**Figure 4:**
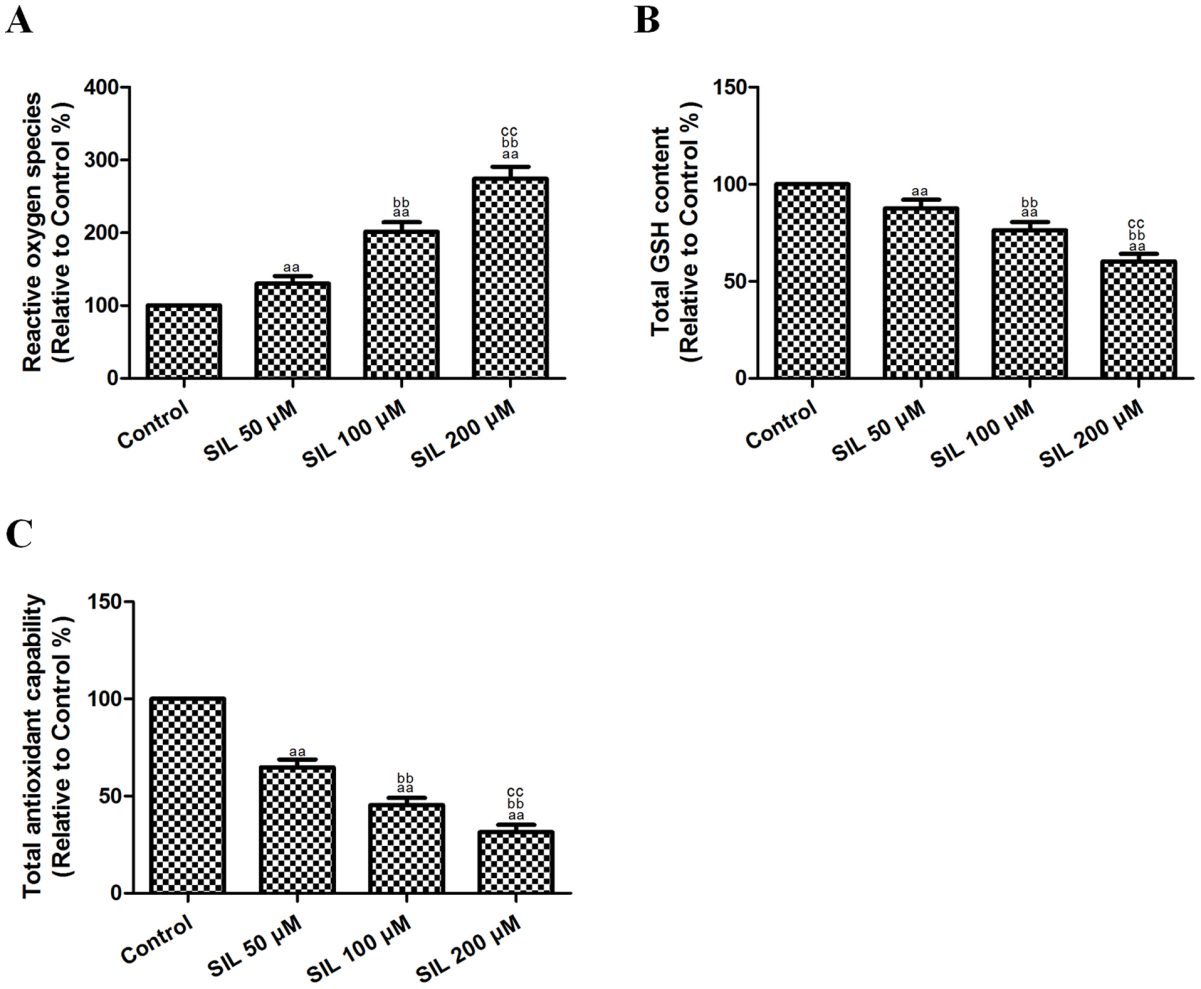
Effects of SIL treatment on ROS generation, GSH levels, and T-AOC in HCC cells (24 h). **A.** ROS concentrations are shown. The fluorescence intensity in the control group was defined as 100%. **B.** Intracellular GSH levels are shown. GSH levels in the control group were defined as 100%. **C**. T-AOCs are shown. Total cellular antioxidant capacity in the control group was defined as 100%. The results are expressed as the means ± SEM, n = 6. ^aa^P<0.01, compared with the control group, ^bb^P<0.01, compared with the 50 µM SIL-treated group, and ^cc^P<0.01, compared with the 100 µM SIL-treated group. SIL, silybin. ROS, reactive oxygen species. GSH, glutathione. T-AOC, total antioxidant capability.

To determine whether SIL causes intracellular oxidation, we used the specific oxidation-sensitive fluorescent dye DCFH-DA, which reveals enhanced fluorescence intensity following the generation of intracellular reactive metabolites. Treatment with SIL (50, 100, or 200 µM) for 24 h induced a dose-dependent increase in ROS generation in HepG2 cells (Figure 4A), with increases of 130.08±10.32%, 201.44±13.09%, and 274.50±16.36% (P<0.01, compared with the control group). Reduced GSH is the major non-protein thiol in cells and is essential for maintaining the cellular redox status. After treatment with SIL (50, 100, or 200 µM) for 24 h, we observed a dose-dependent decrease (87.50±4.50%, 76.33±4.25%, 60.37±3.82%) in intracellular GSH levels (P<0.01, compared with the control group, Figure 4B). SIL treatment also decreased T-AOC in HepG2 cells to 64.83±4.02%, 45.29±3.75%, and 31.43±3.80% (P<0.01, compared with the control group). These results supported the notion that SIL treatment affected the cellular redox status.

### Effects of SIL Treatment on Pro-survival factors and on Proteins in the Notch Signaling and Mitochondrial Apoptotic Pathways in HCC Cells

**Figure 5:**
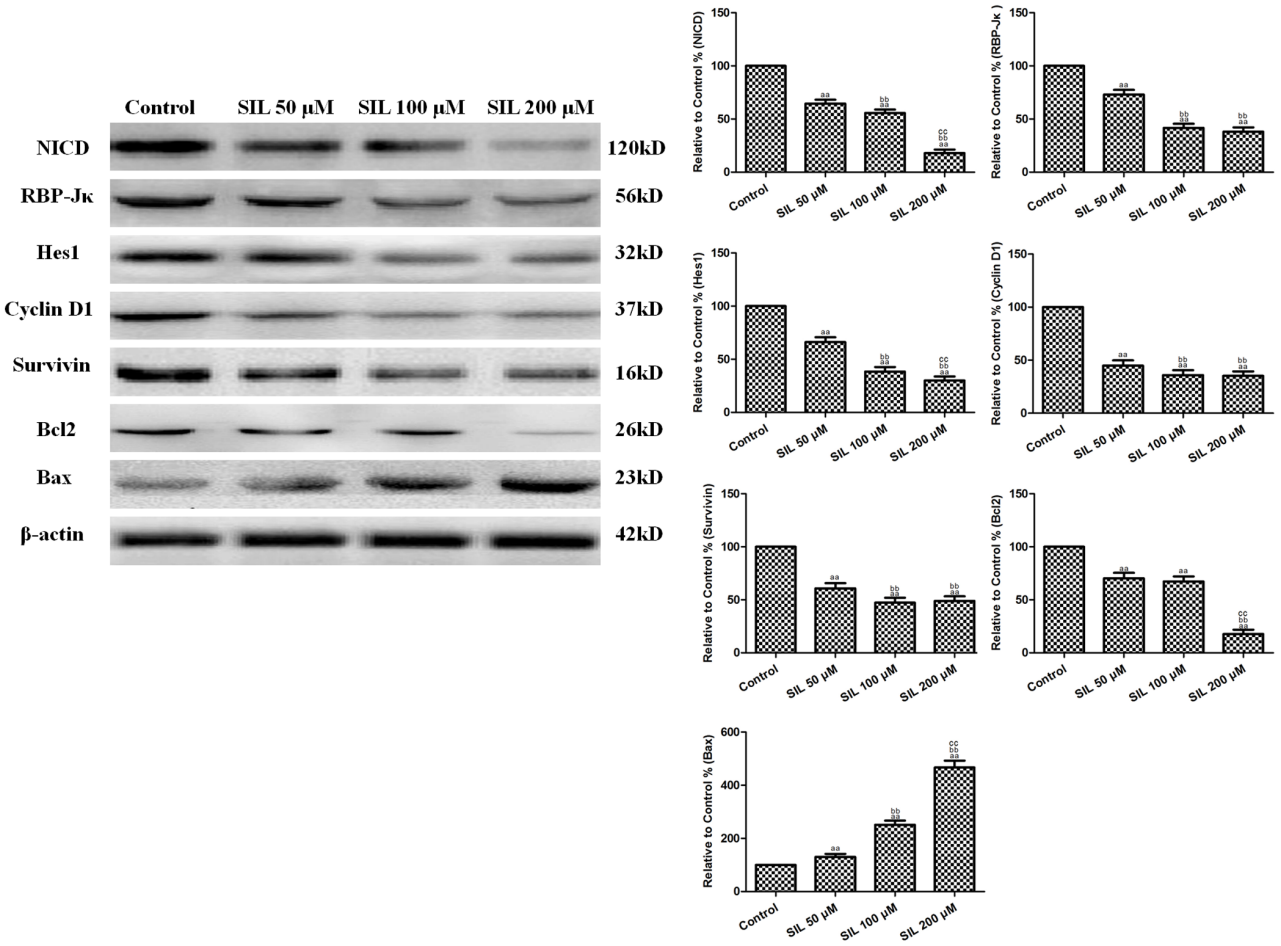
Effects of SIL treatment on the Notch signaling pathway, mitochondrial apoptotic pathway, and pro-survival factors in HCC cells (24 h). Representative Western blot results are shown. SIL treatment decreased Notch1, RBP-Jκ, Hes1, Bcl2, cyclin D1, and survivin expression, and increased Bax expression. The results are expressed as the means ± SEM, n = 6. ^aa^P<0.01, compared with the control group, ^bb^P<0.01, compared with the 50 µM SIL-treated group, and ^cc^P<0.01, compared with the 100 µM SIL-treated group. SIL, silybin.

In order to explore the role of Notch signaling in the antitumor activity of SIL, Notch-related molecules were detected by Western blot analysis. The results of the Western blot analyses revealed that SIL decreased NICD protein levels in HepG2 cells (P<0.01, compared with the control group, Figure 5). We then determined whether decreases in NICD protein levels could attenuate Notch signaling by measuring the expression of the key transcription factor RBP-Jκ and its downstream target, Hes1. Similar to the NICD protein, SIL treatment reduced RBP-Jκ and Hes1 expression (P<0.01, compared with the control group). The results of previous studies have indicated that the activation of Notch signaling can induce the expression of multiple targets that are involved in cellular proliferation, such as cyclin D1 and survivin. In the present study, we also found that SIL treatment decreased the expression of cyclin D1 and survivin (P<0.01, compared with the control group). In addition, the expression levels of proteins related to the mitochondrial apoptotic pathway were altered following SIL treatment: Bax was upregulated, and Bcl2 was downregulated (P<0.01, compared with the control group). These data indicated that the mitochondrial apoptotic pathway was activated.

### Effects of Combined SIL Treatment and Notch1 siRNA Transfection on Cell Viability and Notch Signaling in HCC Cells

**Figure 6:**
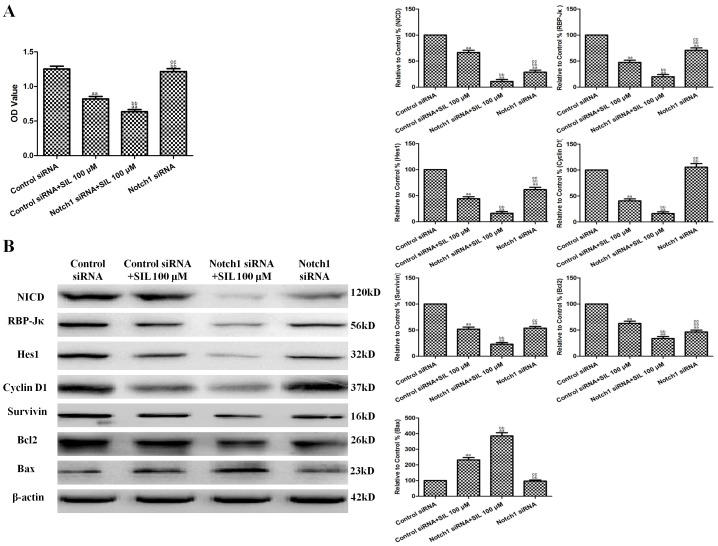
Effects of combined SIL treatment and Notch1 siRNA transfection on cell viability and Notch1 signaling in HCC cells. **A.** Viability is expressed as OD values. **B.** Representative Western blot results are shown. The results are expressed as the means ± SEM, n = 6. ^aa^P<0.01, compared with the control group, ^bb^P<0.01, compared with the 100 µM SIL-treated group, and ^cc^P<0.01, compared with the Notch1 siRNA-transfected +100 µM SIL-treated group. SIL, silybin. OD, optical density.

Notch siRNA was used to explore the effect of the downregulation of Notch signaling on the antitumor activity of SIL in vitro. Notch1 siRNA transfection significantly decreased NICD, RBP-Jκ, and Hes1 levels in HepG2 cells (P<0.01, compared with the control siRNA-transfected group, Figure 6B). Notch1 siRNA also slightly decreased the viability of HepG2 cells, but this difference was not significant (P>0.05, compared with the control siRNA-transfected group, Figure 6A). The combination of Notch1 siRNA transfection and SIL treatment (100 µM for 24 h) significantly decreased cell viability (P<0.01, compared with the SIL-treated or Notch1 siRNA-transfected groups, Figure 6A). In addition, Bax was further upregulated by SIL treatment and Notch1 siRNA co-treatment, while Bcl2, cyclin D1, and survivin were further downregulated (P<0.01, compared with the SIL-treated or Notch1 siRNA-transfected groups, Figure 6B).

### Effects of Combined SIL and Jagged1 Protein Treatment on Cell Viability and Notch Signaling in HCC Cells

**Figure 7:**
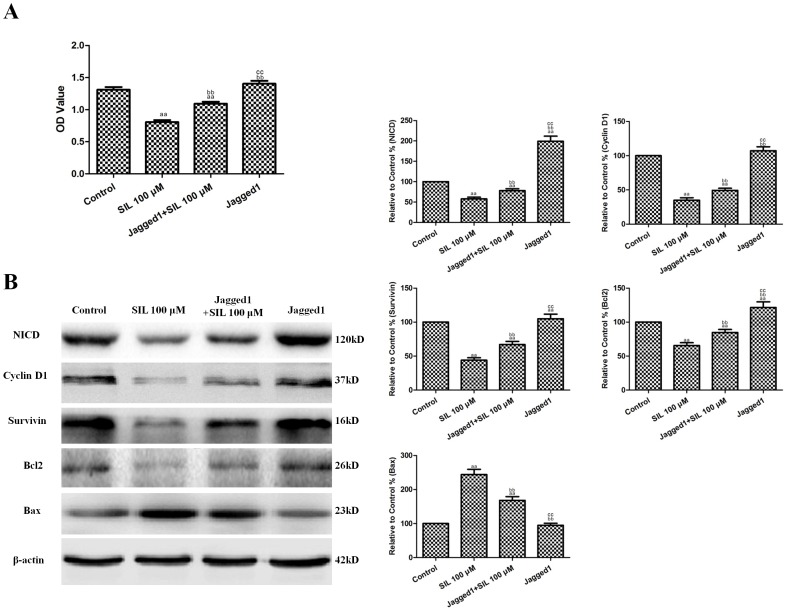
Effects of combined SIL and Jagged1 protein treatment on cell viability and Notch1 signaling in HCC cells. **A.** Viability is expressed as OD values. **B.** Representative Western blot results are shown. The results are expressed as the means ± SEM, n = 6. ^aa^P<0.01, compared with the control group, ^bb^P<0.01, compared with the 100 µM SIL-treated group, and ^cc^P<0.01, compared with the Jagged1+100 µM SIL-treated group. SIL, silybin. OD, optical density.

Recombinant human Jagged1 protein was used to explore the effect of the upregulation of Notch signaling on the antitumor activity of SIL in vitro. HepG2 cells were randomly divided into control, SIL, Jagged1+SIL, and Jagged1 groups (n = 6). The concentration and duration of Jagged1 that effectively increased NICD expression without affecting cell viability (1 µg/mL for 36 h) was selected based on our preliminary experiments. Jagged1 protein pretreatment significantly increased NICD expression in HepG2 cells (P<0.01, compared with the control group, Figure 7B). Jagged1 also slightly increased the viability of HepG2 cells, but this difference was not significant (P>0.05, compared with the control group, Figure 7A). The combination of Jagged1 (1 µg/mL for 36 h) and SIL treatment (100 µM for 24 h) significantly increased cell viability (P<0.01, compared with the SIL-treated group, Figure 7A). In addition, Bax was further downregulated by SIL treatment and Jagged1 co-treatment, while Bcl2, cyclin D1, and survivin were further upregulated (P<0.01, compared with the SIL-treated groups, Figure 7B).

### Effects of SIL on Tumor Xenografts in vivo

**Figure 8:**
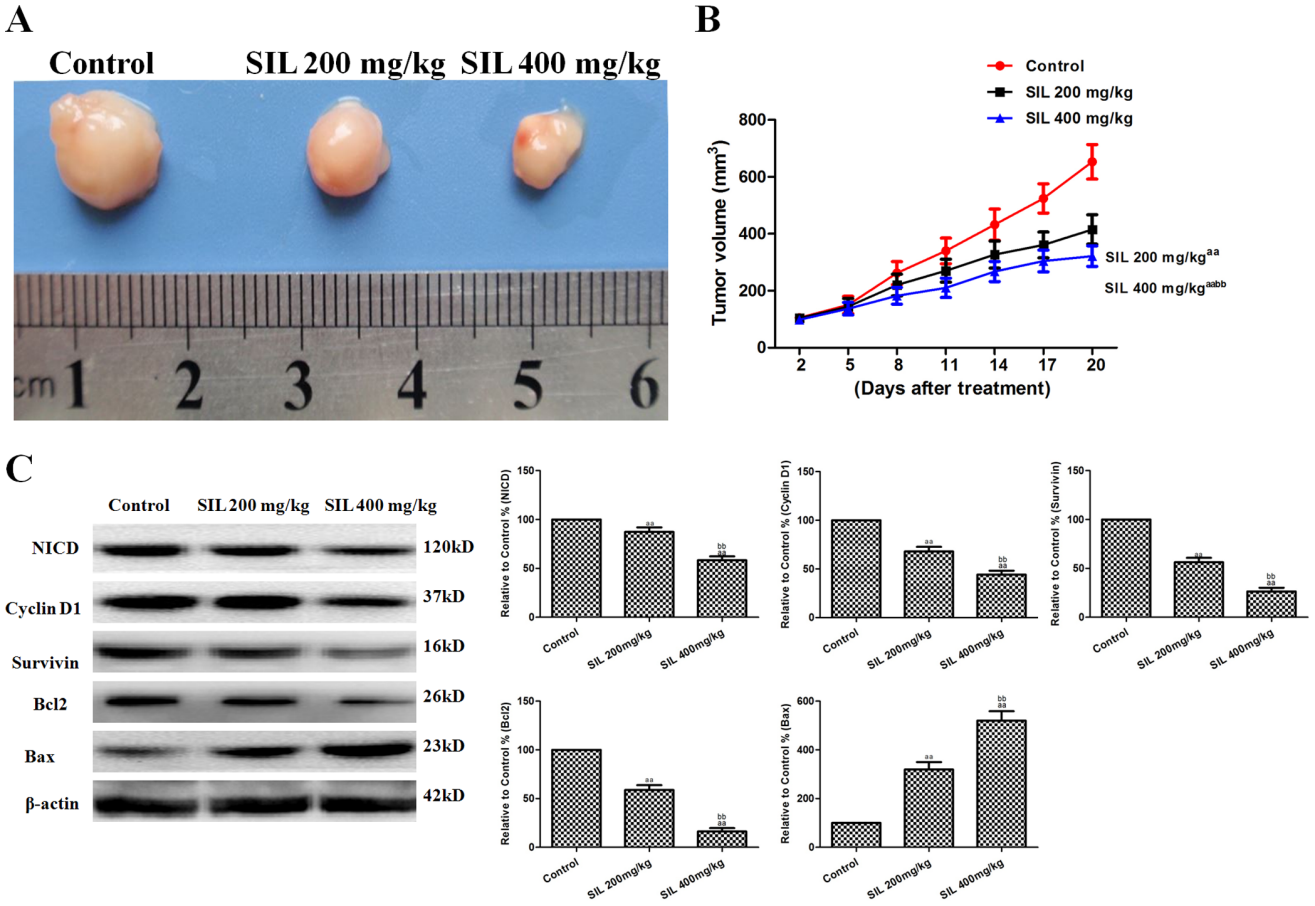
Effects of SIL on HepG2 tumor xenografts in vivo. Photographs showingtumor xenograft morphologies in the various groups. **B.** A tumor growth curve was drawn from the tumor volumes and the treatment duration. **C.** Representative Western blot results are shown. The results are expressed as the means ± SEM, n = 6. ^aa^P<0.01, compared with the control group; ^bb^P<0.01, compared with the SIL200 mg/kg group. SIL, silybin.

Further in vivo tumor xenograft experiments were carried out to verify our previous in vivo experiments. To determine whether SIL could inhibit tumor growth in animals, we established HepG2 xenografts in athymic nude mice. We found that mice in all treatment groups developed subcutaneous tumors. As shown in Figures 8A and 8B, SIL treatment (200 or 400 mg/kg) significantly inhibited tumor growth (P<0.01, compared with the control group). Additionally, Western blot analysis showed that SIL treatment induced a dose-dependent downregulation of NICD, cyclin D1, and survivin (P<0.01, compared with the control group; Figure 8C). Additionally, the mitochondrial apoptotic pathway-related protein Bax was upregulated by SIL treatment, while Bcl2 was downregulated (P<0.01, compared with the control group; Figure 8C).

### Effects of SIL Combined with DAPT on Tumor Xenografts in vivo

**Figure 9:**
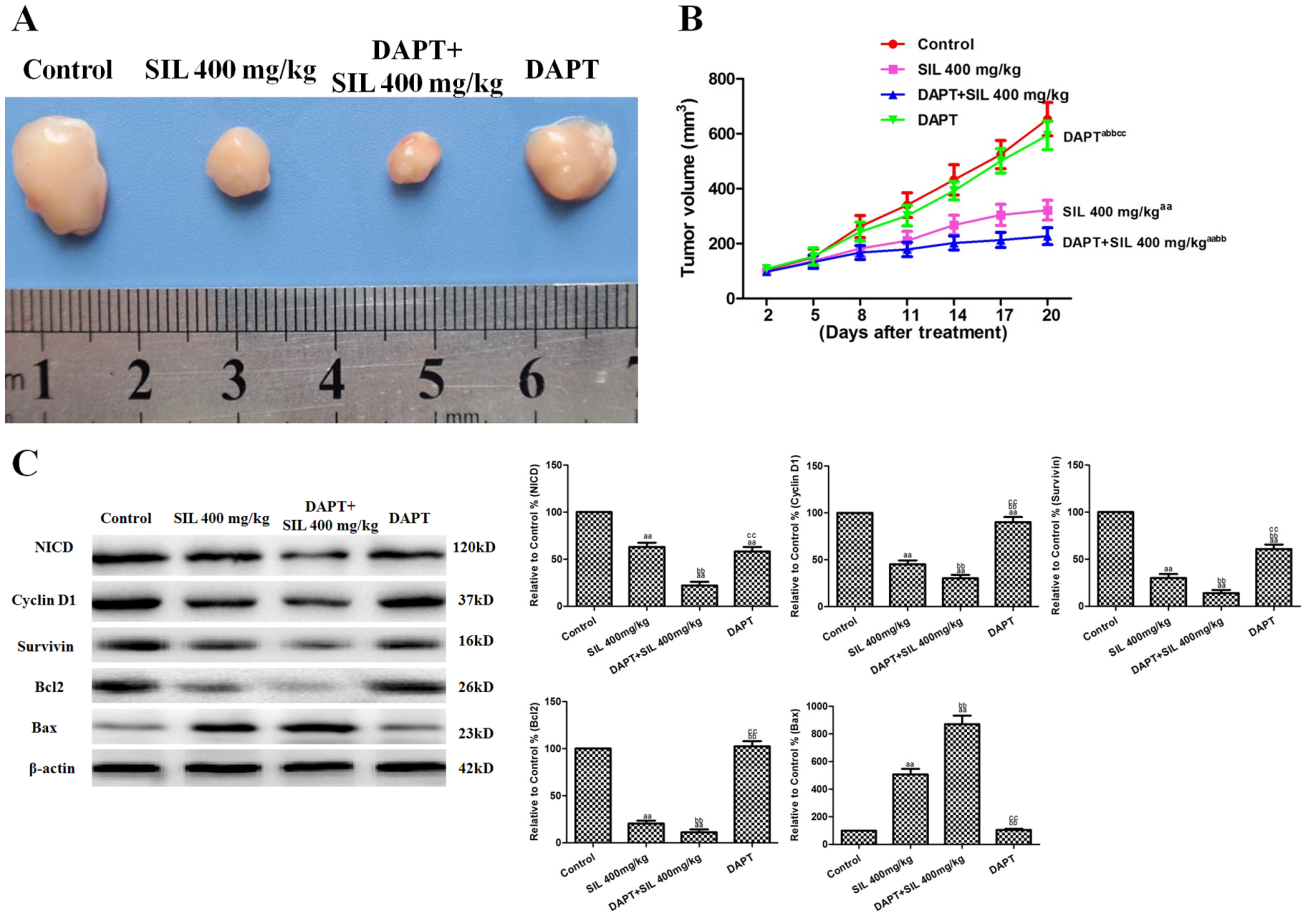
The effects of combined SIL and DAPT on HepG2 tumor xenografts in vivo. Photographs showing tumor xenograft morphologiesinthe different groups. **B.** A tumor growth curve was drawn from the tumor volumes and the treatment duration. **C.** Representative Western blot results are shown. The results are expressed as the means ± SEM, n = 6. ^a^P<0.05, compared with the control group; ^aa^P<0.01, compared with the control group; ^bb^P<0.01, compared with the SIL 400 mg/kg group; ^cc^P<0.01, compared with the DAPT+SIL 400 mg/kg group. SIL, silybin.

DAPT was used to explore the effect of downregulation of Notch signaling on the antitumor activity of SIL in vivo. As shown in Figures 9A and 9B, treatment with SIL or DAPT alone significantly inhibited tumor growth (P<0.01 or P<0.05, compared with the control group). The combination of SIL and DAPT further inhibited tumor growth (P<0.01, compared with the SIL or DAPT groups). Western blot analysis showed that SIL and DAPT co-treatment further decreased NICD, cyclin D1, and survivin expression (P<0.01, compared with the SIL and DAPT groups; Figure 9C). Additionally, Bax was further upregulated by the SIL and DAPT co-treatment, while Bcl2 was further downregulated (P<0.01, compared with the SIL and the D groups; Figure 9C).

## Discussion

SIL is a flavonolignan and the major active constituent of silymarin, a complex mixture of flavonolignans and polyphenols that was extracted from milk thistle seeds. The German Commission E has recommended its use for dyspeptic complaints and liver conditions, including toxin-induced liver damage and hepatic cirrhosis, and as a supportive therapy for chronic inflammatory liver conditions [18]. SIL has been shown to suppress the proliferation of various types of cancer cells, including lung, colorectal, breast, prostate, brain, ovarian and kidney cancers [6]–[10]. Various molecules and signaling pathways are involved in the antitumor effects of SIL, including vascular endothelial growth factor (VEGF) receptor signaling [5], NF-κB signaling [7], extracellular signal-regulated kinase signaling (ERK) [9], protein kinase B signaling (Akt) [9], and signal transducers and activators of transcription (STATs) signaling [19]. However, the effects of SIL on HCC and the mechanisms responsible for these effects are not fully understood. In the present study, SIL treatment resulted in a dose- and time-dependent inhibition of cell viability as well as the induction of apoptosis in HCC HepG2 cells. SIL also significantly blocked HepG2 cell adhesion and migration, both of which are major events that determine tumor metastatic potential. Additionally, SIL treatment significantly inhibited tumor growth in HepG2 xenografts in athymic nude mice.

Oxidative stress refers to an imbalance between pro-oxidant and anti-oxidant factors, which are controlled by multiple components. Oxidative stress may lead to cellular damage. ROS play a key role in oxidative stress and are generated as by-products of cellular metabolism, primarily in the mitochondria [20]. Elevated levels of mitochondrial ROS have been shown to be sufficient to trigger apoptosis [21]. In the present study, the apoptotic effects of SIL in HepG2 cells were associated with rapid increases in the levels of intracellular ROS. Additionally, SIL-induced ROS generation was associated with significant depletions of intracellular GSH, which is a major non-protein cellular antioxidant that can eliminate intracellular ROS [2], [22]. The degree of exposure to ROS and perturbations in the GSH redox balance play critical roles in determining whether cells undergo pro-survival or pro-death responses [3]. Tumor cells are significantly more sensitive to changes in the levels of GSH because tumor cells have heightened basal levels of ROS-mediated signals, which contribute to their increased rates of growth, metabolism and proliferation. Therefore, tumor cells may be more vulnerable to oxidative stress [20]. In the present study, T-AOC was remarkably decreased in SIL-treated HepG2 cells. SIL may have exhausted the total cellular antioxidant capacity and increased the ROS levels beyond a threshold, which may have contributed to the induction of apoptosis in HepG2 cells.

Studies have shown that components of the Notch pathway are overexpressed during HCC progression, as observed for other genes that are known to be involved in the survival of cancer cells [16], [23], [24]. Ahn and colleagues found that Notch1 and Notch4 are markers for poor prognoses during hepatocellular carcinoma [16]. Dill and colleagues confirmed that constitutive Notch2 signaling induces hepatic tumors in mice [23]. Downregulating Notch1 may be an effective approach to inactivating Snail/E-cadherin by regulating cyclooxygenase-2, which results in the inhibition of HCC cell invasion and migration [24]. The overexpression of the key transcription factor RBP-Jκ and its downstream target Hes1 has also been previously reported in HCC [15], [25]. Our results indicate that SIL treatment decreased NICD, RBP-Jκ, and Hes1 in HepG2 cells. When combined with Notch1 siRNA in vitro or DAPT in vivo, silybin further decreased the viability of HCC cells and/or inhibited tumor growth. Additionally, recombinant Jagged1 protein (a known Notch ligand in vitro) effectively attenuated the antitumor activity of SIL. These data suggest that the antitumor activity of SIL in HCC cells is exerted in part through the inhibition of the Notch signaling pathway.

The ability to induce cellular apoptosis is an important property of many candidate anticancer drugs [21]. Apoptosis is a tightly regulated process that involves at least one of the caspase-dependent signaling pathways, i.e., the cell death receptor pathway or the mitochondrial pathway. In the mitochondrial pathway, a variety of death signals trigger the release and translocation of several pro-apoptotic proteins from the mitochondria to the cytosol. Among the numerous factors known to modulate apoptosis in cancer cells, the proteins of the Bcl2 family are considered to be the main regulators of apoptosis. Bcl2 is an anti-apoptotic protein, whereas Bax is a crucial pro-apoptotic and tumor suppressor protein. The ratio of anti-apoptotic to pro-apoptotic molecules, such as the Bcl2/Bax ratio, indicates the threshold sensitivity of cells to the induction of apoptosis via the intrinsic pathway [20]. The activation of caspases is a pivotal step in the apoptotic process and is triggered by signals from death factors, mitochondrial alterations or DNA damage due to external and/or internal insults [26]. Regardless of whether apoptosis is mediated via the cell death receptor pathway or the mitochondrial pathway, both of the pathways ultimately activate caspase3, which in turn induces DNA fragmentation, the characteristic morphological change that is associated with apoptotic cells. Caspase3 activation indicates a key and irreversible point in the induction of apoptosis [27]. Our results indicated that SIL treatment not only downregulated Bcl2 protein expression but also upregulated Bax protein expression and caspase3 activity in HepG2 cells. These results indicated that SIL-induced apoptosis occurred via the mitochondrial pathway and verified the results of previous studies that indicated that the inhibition of Notch signaling is associated with the induction of the mitochondrial apoptotic pathway in human cancer cells [28], [29].

The results of the present study also indicate that the viability of HepG2 cells was decreased by the use of Notch1 siRNA (in vitro) or DAPT (in vivo) in combination with SIL treatment. These decreases were associated with decreases in NICD, RBP-Jκ, Hes1, and Bcl2 expression, as well as with increases in Bax expression. These results indicate that the inhibition of Notch signaling may be a novel means both of enhancing the effects of chemotherapy and of delaying chemoresistance in patients with cancer. In addition, Notch1 siRNA or DAPT in combination with SIL treatment further suppressed the expression of cyclin D1 and survivin, two pro-survival factors that are targets of Notch1 and have been reported to play roles in sensitization to anticancer drugs [15], [30], [31]. These results suggest that the inhibition of Notch signaling may sensitize HCC cells to SIL treatment by preventing the activation of pro-survival pathways. The concentration of DAPT (10 mg/kg) that did not affect tumor volume significantly (compared with the control group) was selected based on our preliminary experiments. As expected, DAPT treatment alone significantly decreased NICD expression (compared with the control group); however, DAPT alone only slight affected Cyclin D1, Bcl2, and Bax gene expression, while SIL had very potent effects on tumor suppression and on the expression of these proteins. These results indicate that Notch signaling is not the only pathway that mediates the function of SIL.

In conclusion, these experiments provide mechanistic evidence that the Notch pathway is inhibited in HCC cells in response to SIL treatment. The downregulation of Notch signaling sensitizes HCC cells to SIL treatment, and upregulation of Notch signaling desensitizes HCC cells to SIL treatment. Additionally, this potentiation of chemosensitivity by Notch inhibition may be related to the downregulation of pro-survival pathways. Therefore, we propose that the inhibition of Notch signaling may be a novel strategy that can be used to prevent the induction of cancer survival mechanisms in advanced HCC.
